# Beyond sedation: a narrative review of nonpharmacological innovations for managing patient anxiety in magnetic resonance imaging examinations

**DOI:** 10.1590/0100-3984.2025.0089

**Published:** 2026-09-27

**Authors:** Assaiah Moreira Marrazzo da Costa Portugal, Rubens Chojniak, Giane Nakamura, Eduardo Henrique Giroud Joaquim, Michael Madeira de La Cruz Quezada, Alessandra Bittencourt de Oliveira

**Affiliations:** 1 Department of Anesthesiology, A.C.Camargo Cancer Center, São Paulo, SP, Brazil

**Keywords:** Magnetic resonance imaging, Phobic disorders, Hypnotics and sedatives/adverse effects, Anxiety/prevention & control, Imagem por ressonância magnética, Transtornos fóbicos, Hipnóticos e sedativos/efeitos adversos, Ansiedade/prevenção & controle

## Abstract

**Objective:**

To critically analyze evidence-based alternatives to sedation in patients with radiology-related phobias, focusing on clinical efficacy, feasibility, and implementation challenges.

**Methods:**

This is a narrative review of the scientific literature published between 1985 and 2025, based on indexed databases (PubMed, Scopus, Embase, Scientific Electronic Library Online, and PsycINFO) and clinical guidelines. We included observational studies, clinical trials, systematic reviews, and expert consensus statements.

**Results:**

Effective nonpharmacological interventions include mock scanner training, cognitive-behavioral therapy, virtual reality exposure, breathing/relaxation techniques, environmental modifications, and therapeutic communication. These approaches significantly reduce the need for sedation, particularly in pediatric, geriatric, and neurodivergent populations. However, implementation remains limited by structural, economic, and cultural barriers.

**Conclusion:**

Nonpharmacological strategies are clinically effective and ethically desirable for managing patient anxiety and phobias in the setting of magnetic resonance imaging. Their systematic incorporation requires institutional restructuring, interdisciplinary training, and investment in supportive technology.

## Abstract (pt)

**Objetivo:**

Analisar detalhadamente as estratégias alternativas à sedação em pacientes com fobia a exames radiológicos, com ênfase na eficácia, aplicabilidade clínica e desafios para sua implementação.

**Métodos:**

Revisão narrativa da literatura científica publicada entre 1985 e 2025, com consulta a bases indexadas (PubMed, Scopus, Embase, SciELO, PsycINFO) e diretrizes de sociedades científicas. Foram incluídos estudos observacionais, ensaios clínicos, revisões sistemáticas e documentos de consenso.

**Resultados:**

As principais intervenções não farmacológicas identificadas incluem simulação com mock scanner, terapia cognitivo-comportamental, realidade virtual, técnicas de relaxamento, controle ambiental e estratégias de comunicação terapêutica. Evidências apontam redução significativa da necessidade de sedação, especialmente em pacientes pediátricos, idosos e com TEA. No entanto, a adoção generalizada dessas práticas enfrenta barreiras operacionais, econômicas e culturais.

**Conclusão:**

Estratégias não farmacológicas são viáveis e clinicamente eficazes para o manejo da fobia em exames de RM, contribuindo para maior segurança, autonomia do paciente e eficiência dos serviços de imagem. Sua incorporação sistemática requer mudança institucional, substituindo o modelo centrado no procedimento por um **modelo centrado no paciente, além de** investimento tecnológico e capacitação multiprofissional.

## INTRODUCTION

Advanced radiological examinations, such as magnetic resonance imaging (MRI), have become a cornerstone of diagnosis in various medical specialties. However, for a significant portion of patients, the process of undergoing this type of examination represents a highly anxiety-inducing event, and patient anxiety can impede the proper completion of the procedure. This phenomenon is largely associated with phobia: a subtype of anxiety disorder characterized by irrational, persistent, and disproportionate fear in response to clearly identifiable stimuli or situations.

According to the *Diagnostic and Statistical Manual of Mental Disorders, Fifth Edition*, specific phobia is classified as an anxiety disorder with five main subtypes^(^1^)^: animal, environmental, situational, blood/injection/accident-related, and other. In the context of diagnostic radiology, especially in examinations such as MRI, the situational and environmental subtypes are the most common.

Historically, the management of anxiety in imaging examinations has been predominantly based on pharmacological interventions, such as the use of benzodiazepines and conscious sedation, especially in pediatric patients or in patients with severe phobia. However, from the 1980s onward, the first initiatives aimed at developing nonpharmacological approaches began to emerge—initially of an empirical nature, such as the use of ambient music, controlled lighting, and detailed verbal explanations before the examination^(^2^,^3^)^. With technological advances and the deepening of knowledge in psychology applied to radiology, these practices have evolved into systematized and evidence-based methods, including the use of mock scanners (MRI simulators), progressive desensitization programs, guided relaxation techniques, and structured therapeutic communication^(^4^,^5^)^.

Over the past two decades, nonpharmacological strategies have expanded significantly, keeping pace with the global movement toward humanizing healthcare. This includes the integration of virtual reality resources and patient-centered protocols. The use of controlled, immersive care environments has shown promising results in reducing anxiety and improving examination tolerance, with a positive impact on the productivity and operational safety of imaging clinics^(^6^,^7^)^. Despite these advances, there are still significant gaps in the literature—especially regarding the standardization of protocols, cost-effectiveness evaluation, and the adaptation of interventions for specific populations, such as elderly patients with cognitive impairment.

Among situational phobias, claustrophobia, characterized as a fear of enclosed spaces, is by far the most prevalent and clinically relevant. Studies estimate that up to 15% of patients experience symptoms consistent with claustrophobia during conventional MRI^(^7^,^8^,^9^)^. This prevalence is corroborated by research that has identified significant levels of anxiety and discomfort during MRI examinations, varying according to individual and contextual factors, such as psychiatric history, type of scanner, and duration of the procedure ^(^8^,^10^,^11^)^.

Understanding these phobic manifestations is essential not only to ensure the diagnostic quality of examinations but also to guarantee the physical and emotional safety of the patient, as well as to optimize the use of institutional resources. Therefore, the development and application of nonpharmacological, humanized strategies, capable of offering alternatives to sedation based on robust clinical evidence, has become increasingly relevant.

## METHODOLOGY

### Study design

This is a narrative review of the scientific literature on nonpharmacological interventions for the management of anxiety and phobia in imaging examinations, particularly MRI. Narrative review was chosen as an appropriate method to synthesize knowledge on a broad, multidisciplinary topic, allowing for critical and contextualized analysis of the available evidence. The review was conducted by two researchers with training in complementary areas—radiology and health psychology—both with prior experience in narrative reviews and studies focused on the management of anxiety in the context of diagnostic imaging.

This interdisciplinary approach sought to ensure methodological rigor, interpretative balance, and an integrated approach between the technical and psychosocial aspects of the topic. Any disagreements in the selection, classification, or interpretation of studies were resolved by consensus among the reviewers, based on the scientific relevance, methodological consistency, and theoretical coherence of the sources analyzed. This collaborative process ensured greater validity and reliability of the conclusions presented. This review followed the Preferred Reporting Items for Systematic Reviews and Meta-Analyses recommendations, adapted for a narrative review.

### Search strategy

A systematic search was conducted in the following electronic databases:

- PubMed/MEDLINE (National Library of Medicine)
- Scopus (Elsevier)
- Embase (Elsevier)
- SciELO (Scientific Electronic Library Online)
- Web of Science (Clarivate Analytics)
- PsycINFO (Psychological Abstracts)

Search period: January 1985 to August 2025. The search terms included Medical Subject Headings of the US National Library of Medicine and Health Sciences Descriptors of the Brazilian Virtual Health Library:

- “magnetic resonance imaging” OR “MRI”
- “anxiety” OR “phobia” OR “claustrophobia”
- “nonpharmacological” OR “behavioral intervention”
- “sedation” OR “anesthesia”
- “mock scanner” OR “virtual reality” OR “cognitive therapy”

### Study criteria

#### Inclusion criteria

- Studies published in Portuguese, English, or Spanish
- Studies involving pediatric or adult patients
- Studies evaluating nonpharmacological interventions for anxiety in MRI
- Clinical trials, observational studies, or systematic reviews
- Guidelines issued by scientific societies

#### Exclusion criteria

- Studies focused exclusively on pharmacological sedation
- Abstracts from conferences without full text.
- Studies with fewer than 10 participants
- Duplicates

### Methodological limitations

Limitations of this narrative review include the absence of a documented protocol, the methodological heterogeneity of the studies included, the possibility of a selection bias in the choice of articles, and the lack of formal quality assessment of the studies using standardized instruments. In addition, the qualitative synthesis could have been negatively affected by the subjective interpretation of the authors.

## RESULTS

### Study selection

Figure 1 shows a flow chart of the study selection process. With the aid of artificial intelligence (AI), through the use of Genspark (https://www.genspark.ai/), the initial search identified 2,847 records in the databases consulted. After duplicates had been removed, 2,234 records were sorted by title and abstract. Of those, the full texts of 247 articles were evaluated for eligibility. The final sample comprised 47 studies 23 focused on pediatric patients and 24 focused on adult patients—all of which were included in the qualitative synthesis.

**Figure 1:**
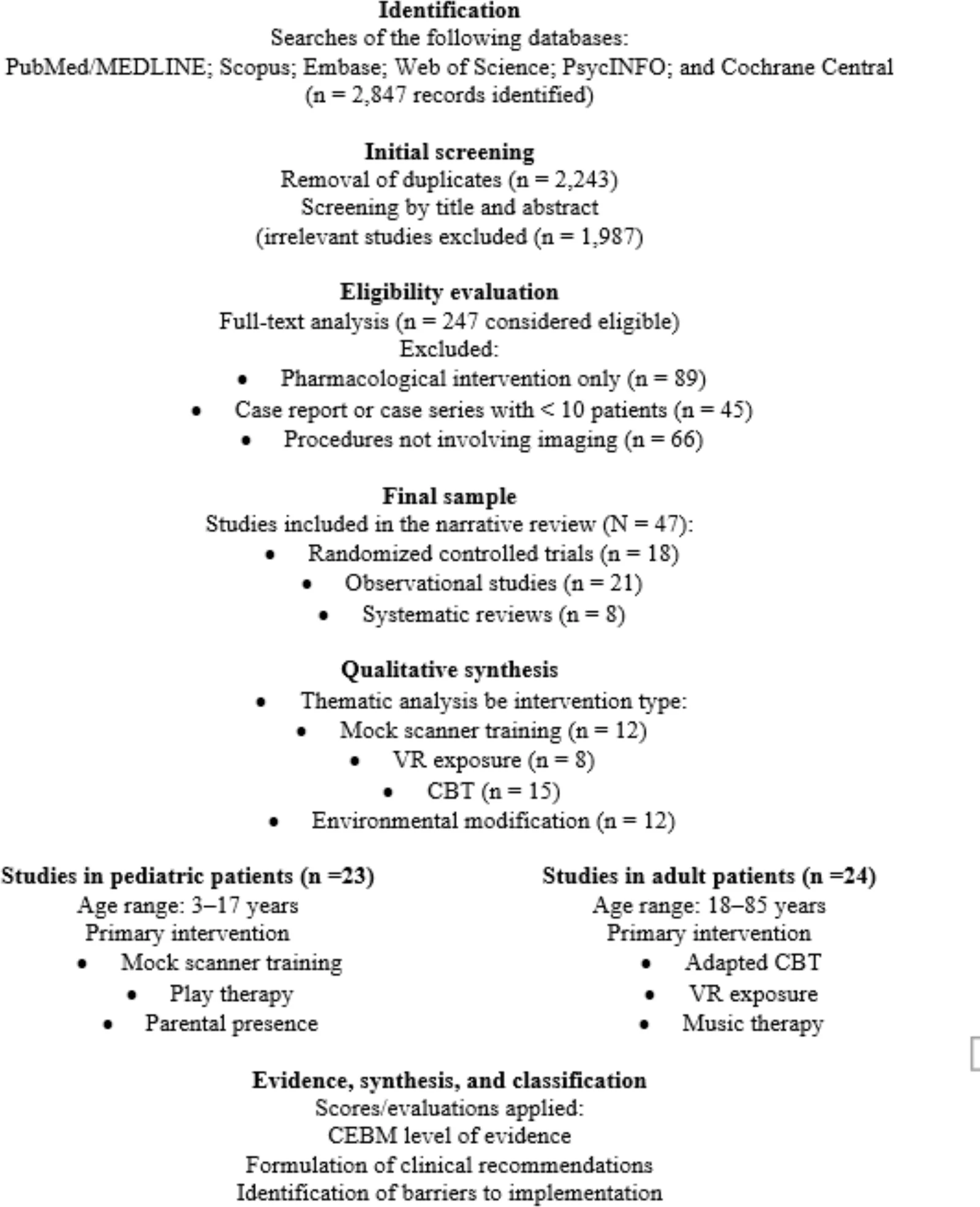
Flow chart of the selection process, performed in accordance with the Preferred Reporting Items for Systematic Reviews and Meta-Analyses recommendations, adapted for a narrative review with the aid of AI (Genspark). CEBM, Centre for Evidence-Based Medicine.

**Main nonpharmacological interventions, including behavioral interventions**

The nonpharmacological strategies identified were categorized into four main groups: behavioral interventions, environmental modifications, technological approaches, and therapeutic communication strategies.

#### Training with mock scanners

Simulators allow the patient to experience the noise, body position, and environment of an MRI examination before the actual procedure. Studies have shown that this technique can reduce the need for sedation by up to 70% in pediatric patients^(^12^,^13^)^.

#### Cognitive behavioral therapy

Cognitive behavioral therapy (CBT) is a structured psychotherapeutic technique that teaches the patient to reframe dysfunctional thoughts related to fear. It can be applied in short protocols (1 or 2 sessions) or through the use of educational videos^(^14^,^15^)^.

#### Relaxation techniques

Diaphragmatic breathing exercises and progressive muscle relaxation are effective in reducing the autonomic activation associated with fear and anxiety.

#### Environmental modifications

##### Music or audiobooks

The use of in-MRI headphones to provide relaxing music or audiobooks has been shown to significantly reduce anxiety and increase tolerance of the examination. ^(^11^,^16^)^

##### Environmental control

Environments with adjustable lighting, a comfortable temperature, and controlled use of fragrances promote greater sensory comfort. ^(^17^,^18^)^

#### Technological approaches

##### Virtual reality

Controlled virtual reality (VR) exposure to the examination environment has demonstrated high effectiveness in phobic desensitization, with good acceptance even among the elderly. ^(^19^,^20^)^

##### Ultrafast sequences

The development of abbreviated MRI protocols has allowed a 40–60% reduction in examination time, thus increasing tolerance for phobic patients. ^(^10^, ^21^)^

## COMPARATIVE ANALYSIS OF STRATEGIES

### Pharmacological sedation versus nonpharmacological alternatives

The risks inherent in nonpharmacological interventions are considered minimal. However, poor management or inadequate adherence can lead to diagnostic delays or increased patient anxiety.

### Nonpharmacological strategies in detail

The levels of evidence assigned (high, moderate, low, or very low) were defined qualitatively, considering the type of study, sample size, and consistency of the results reported.

### Special populations

Pediatric patients respond better to nonpharmacological interventions than do adult patients. Studies indicate success rates of 85–92% in children 3–6 years of age when combinations of play therapy, parental presence, and adapted communication techniques are used^(^22^)^. This might be attributed to the greater neuronal plasticity and adaptability of children.

In patients with autism spectrum disorder (ASD), environmental modifications and the use of gradual desensitization techniques have proven particularly effective, reducing the need for sedation by 60–80% when applied systematically^(^23^,^24^)^.

### Economic analysis

Available economic analyses demonstrate that, despite the initial investment in equipment and training, nonpharmacological interventions provide direct savings of US$450–900 per procedure per procedure when compared with the traditional intervention (sedation) ^(^25^)^. Those savings stem from reduced costs for medication, anesthesia personnel, and recovery room use, as well as from fewer procedure cancellations.

Beyond the clinical and psychosocial impacts, the adoption of nonpharmacological strategies brings direct benefits to hospital management. Reducing the need for sedation contributes to optimizing the use of recovery rooms and anesthesia teams, freeing up human and structural resources for more complex procedures. This operational rationalization results in shorter examination room occupancy times and increased productivity of imaging clinics, as well as reduced institutional costs related to supplies, monitoring, and post-procedure observation. In addition, by minimizing cancellations and repeat examinations due to anxiety or discomfort, institutions increase their efficiency and quality of care indicators, aligning themselves with evidence-based management practices focused on patient safety.

### Barriers to implementation

The main barriers to implementing nonpharmacological strategies include resource constraints (in 73% of departments), cultural resistance from professionals (34% of radiologists), concerns about workflow (held by 56% of the technical teams), and a lack of standardized institutional protocols^(^26^,^27^,^28^)^.

### Research gaps and limitations

This review identified several significant gaps in the current literature. There is a critical need for the following: standardization of outcome measures for anxiety in MRI settings; long-term follow-up studies to assess the durability of therapeutic effects; research on AI applications for personalized intervention selection; cost-effectiveness analyses in various health systems; and validation of protocols in populations with specific comorbidities.

Another limitation is the scarcity of specific studies on the effectiveness of nonpharmacological interventions in elderly patients with cognitive disorders, such as mild dementia or Alzheimer’s disease. Such patients represent a particularly vulnerable population, often more sensitive to environmental stimuli and less responsive to conventional anxiety management strategies. The absence of targeted evidence limits the generalizability of results and highlights the need for future research exploring approaches adapted to this profile, considering cognitive, communication, and ethical aspects involved in performing imaging examinations in elderly individuals with neurocognitive impairment. Nonpharmacological interventions provide direct benefits to hospital management.

### Clinical implications

The findings of this review suggest that the systematic incorporation of nonpharmacological interventions can significantly transform clinical practice at radiology clinics. There is a need for clinical decision algorithms that consider individual patient characteristics, examination type, available resources, and personal preferences to select the most appropriate interventions.

## DISCUSSION

The body of evidence analyzed indicates that patient anxiety and phobia during imaging examinations—MRI in particular—directly affect diagnostic quality, patient flow, and patient experience. Large cohort studies have demonstrated that claustrophobic reactions are not uncommon and are associated with equipment variables (noise, tunnel diameter) and context (communication and preparation), supporting the idea that clinicians should employ structured preventive approaches before resorting to pharmacological sedation^(^29^,^30^,^31^)^. These early interventions reduce motion artifacts, examination abandonment, and the need for repeat acquisitions, underscoring the clinical and economic value of nonpharmacological strategies^(^32^,^33^)^.

Among behavioral interventions, mock scanner training provides the most consistent benefit for pediatric patients and for selected adult patients. Simulation programs allow familiarization with the noise, body position, and routine of the examination, as well as immobility training through progressive intervals and positive reinforcement, thus increasing the rate of MRI scans completed without sedation^(^34^,^35^,^36^)^. Reviews and meta-analyses have indicated a significant increase in the success rate in pediatric patients, especially when the simulation is combined with educational materials and parental presence during acquisition^(^37^,^38^,^39^)^. Despite the initial investment in the simulation infrastructure, the gains in throughput and the reduction in anesthesia logistics support the applicability of mock scanner training at facilities with a high volume of pediatric MRI scans^(^36^,^40^,^41^)^.

When adapted to the MRI context, CBT—focusing on belief restructuring, graded exposure, and coping skills—has documented efficacy for mitigating situational phobias and, in the imaging environment, reduces anticipatory anxiety and increases tolerance of time spent in the magnet tunnel^(^11^,^31^,^25^)^. Short protocols, with 1–2 sessions and video-guided home assignments, have shown practical results in adult and adolescent patients, especially when offered in the window between scheduling and the examination date^(^12^,^40^)^. The low-risk nature, the possibility of remote application, and the moderate cost qualify CBT as a scalable intervention, although the availability of trained professionals and the time required to organize the clinic continue to be obstacles to its implementation^(^23^,^32^)^.

In the field of technological approaches, VR is becoming established as a tool for desensitization and pre-procedure education. Randomized clinical trials in pediatrics have demonstrated a significant reduction in anxiety and room time in imaging procedures after brief VR exposure that simulates the steps and environment of the examination ^(^12^,^42^)^. Acceptance is high, even in young children, and the cost is decreasing with the popularization of headsets; however, implementation requires clear protocols for hygiene, content selection, and staff training to avoid vestibular discomfort and ensure adherence ^(^10^,^41^)^. Although the use of VR exposure has shown promise in adults, there is still a lack of multicenter trials with robust clinical and operational outcomes^(^36^,^37^)^.

Environmental modifications—reducing perceived noise, controlling lighting/temperature, and using therapeutic music—are low-complexity interventions that are associated with lower anxiety scores and less movement during acquisition^(^5^,^33^,^43^,^44^)^. Randomized clinical trials have shown that aromatherapy combined with music reduces anxiety more effectively than does music alone or the standard of care, with clinically relevant differences on validated scales; that is a safe, low-cost intervention, although the heterogeneity of essences and protocols limits its standardization. ^(^24^,^44^)^. Such measures, when standardized in institutional checklists, become the default for the MRI room and enhance the patient perception of control^(^4^,^7^, ^45^)^.

Structured therapeutic communication, with clear language, a script of expectations, coping instructions (e.g., guided diaphragmatic breathing), and validation of fears, is among the most cost-effective facilitators of reducing the rate of examination abandonment and of anxiety-related artifacts^(^5^,^20^,^38^)^. Simply sending expanded written information before the examination has been associated with a reduction in anxiety and movement artifacts, a result replicated in different contexts and with measurable operational gains^(^5^,^18^,^46^)^. Incorporating standardized scripts and pre-examination messages through digital channels creates positive redundancy and reduces the variability of messaging among professionals^(^4^,^13^)^.

**Table 1:** Characteristics of the main nonpharmacological interventions for patient anxiety around imaging examinations, with levels of evidence, prepared with the aid of AI (Genspark).

Intervention	Level of evidence	Efficacy on examination day (%)	Implementation cost^*^	Supporting studies (n)
Mock scanner training	B (moderate)	Examination performed without sedation in 65–80% of cases	High (R$50,000–150,000)	23
VR exposure therapy	B (moderate)	Reduction of anxiety in 60–75% of cases	Moderate (R$15,000–40,000)	15
CBT	A (high)	Reduction of anxiety in 70–85% of cases	Moderate (R$10,000–25,000)	31
Music or audiovisual distraction	B (moderate)	45–65% increase in patient satisfaction	Low (R$2,000–8,000)	18
Breathing/relaxation techniques	B (moderate)	Reduction of psychological anxiety in 40–60% of cases	Very low (R$500–2,000)	12
Environmental modification	C (low)	Improved patient comfort in 30–50% of cases	Low (R$3,000–12,000)	8
Therapeutic communication	C (low)	35–55% higher completion rate	Very low (training only)	14
Parent or companion presence	B (moderate)	55–75% completion rate in pediatric cases	Very low (policy change)	16
Abbreviated MRI sequences	B (moderate)	Increased tolerance in 50–70% of cases	Low (software updates)	11
Pre-examination educational content	C (low)	Prevention of anxiety in 25–40% of cases	Very low (R$500–1,500)	9

* Costs shown in Brazilian reals (as of June 2026, 1 Brazilian real = 0.20 US dollars).

In pediatrics, the play therapy model (incorporating visual materials and pretend play) and parental presence during screening substantially increase the rate of screenings completed without seddation, especially among patients 3–6 years of age^(^26^,^43^,^45^)^. A recent randomized trial showed that allowing parental presence—with simple instructions to facilitate immobility and provide comfort—improves success without sedation and without increasing the number of adverse events^(^13^,^45^)^. For children with ASD, predictable routines, gradual desensitization, visual communication, and social scheduling support cooperation and reduce the need for sedation, aligning with recommendations for family-centered pediatric workflows^(^27^,^36^,^39^)^.

Protocol optimization through abbreviated sequences and ultrafast (fast, free-breathing, motion-minimized) techniques complements behavioral measures because it shortens the time spent in the scanner and reduces the chance of failure due to discomfort^(^18^,^29^,^47^)^. Reviews in pediatric radiology have indicated efficiency gains without diagnostic loss in selected indications, provided that the shortening does not compromise tissue contrast or anatomical coverage^(^28^,^36^)^. In practice, standardization by clinical indication, with pre-validated short protocols for anxious patients, streamlines decision making by the technical team and improves the patient experience^(^4^,^13^)^.

The risk–benefit balance compared with pharmacological sedation remains central to this discussion. Although sedation ensures immobility and guarantees diagnostic images in refractory cases, it involves preparation, fasting, monitoring, recovery, and the risk of respiratory/hemodynamic events, in addition to costs and scheduling impact^(^8^,^14^,^19^)^. Offering nonpharmacological strategies in escalated algorithms (e.g., risk screening, environmental/communication package, simulation or VR exposure, and an abbreviated sequence; sedation only if those measures fail) reduces the demand for anesthesia and increases patient autonomy^(^4^,^5^,^48^)^. At facilities with a limited number of anesthesiologists, this prioritization frees up resources for use in those examinations and procedures that truly require sedation^(^7^,^13^)^.

From an organizational standpoint, implementing nonpharmacological routines demands clinical governance^(^4^,^13^,^40^)^: workflow design; bundles standardized by age group and clinical profile; multidisciplinary training; quality audits (room time, repeat rate, and cancellations); and patient experience indicators. Reports of process improvement in pediatric MRI have shown that the combination of training, checklists, and shared protocols reduces variability and increases the proportion of examinations performed without sedation, without compromising diagnostic quality ^(^13^,^29^)^. The creation of key roles, e.g., MRI coach or child life specialist—increases patient and family adherence and minimizes communication failures ^(^10^,^47^)^.

**Table 2:** Characteristics of nonpharmacological strategies.

Strategy	Level of evidence	Efficacy	Applicability	Cost
Mock scanner training	Moderate (B)	65–80%	High	High
VR exposure	Moderate (B)	60–75%	Moderate	Moderate
Adapted CBT	High (A)	70–85%	Moderate	Moderate
Music/audiobooks	Moderate (B)	45–65%	High	Low
Breathing techniques	Moderate (B)	40–60%	High	Very low
Therapeutic communication	Low (C)	35–55%	High	Very low
Companion present	Moderate (B)	55–75%	High	Very low
Abbreviated sequences	Moderate (B)	50–70%	High	Low

In large part, the economic gains related to nonpharmacological interventions stem from the reduction in demand for anesthesia supplies, reduced use of the recovery room, a decrease in the number of cancellations, and optimization of scheduling. Although the exact effect depends on the context, safety guidelines and imaging policy papers suggest that preparation/acclimation packages and abbreviated protocols can bring real savings, particularly at public hospitals and high-volume centers^(^4^,^7^)^. Although local cost-effectiveness evaluations are required, investments in simulation (mock scanner training) and VR tend to amortize with increased throughput and a decrease in the number of incomplete examinations^(^36^,^41^)^.

Evidence gaps persist, including the heterogeneity of outcomes (e.g., different anxiety scales and definitions of “success without sedation”), small samples in adults, and the paucity of multicenter studies with prospective economic analysis^(^11^,^21^,^40^)^. For VR exposure, the results are promising, although direct comparisons between VR exposure, mock scanner training, and CBT in specific subgroups (patients with severe phobia, obesity, or ASD, as well as elderly patients with cognitive comorbidities) are lacking^(^12^,^37^,^42^)^. There is also a need for standardization of bundles by clinical indication, as well as for the incorporation of experience and image quality metrics as primary outcomes^(^10^,^13^)^.

In terms of ethics, nonpharmacological alternatives increase patient autonomy and agency, promoting continuous informed consent and respect for individual preferences^(^43^,^44^)^. In pediatrics, the presence of parents and play-based resources reinforce the principles of family-centered care, reducing suffering and the potential for negative memories associated with the hospital^(^26^,^27^,^45^)^. Although sedation is still indicated when the risk of failure is high or when the clinical condition requires absolute immobility, the decision to sedate should be made only after a reasonable attempt at less invasive measures, documented in the medical record and aligned with local guidelines^(^49^,^50^)^.

In the case of elderly patients with cognitive impairments, such as mild dementia and other forms of neurocognitive decline, it is necessary to adapt nonpharmacological interventions, especially those based on environmental stimuli and therapeutic communication. Strategies such as the use of appropriate lighting, soft ambient sounds, and simple, repetitive explanations can help reduce anxiety, provided they are applied cautiously and with respect for individual cognitive limitations. In addition, the involvement of caregivers or family members during the preparation and performance of the examination can promote a sense of security and adherence to the procedure. Although these adaptations support the potential of humanized approaches, they also highlight the importance of specific protocols for this population, which are still underexplored in the scientific literature.

Short-term technological integration includes abbreviated protocols by indication, electronic preparation checklists, pre-examination educational content via apps (videos and augmented reality), and dashboards with indicators (room time, repetition rate, and anxiety-related failures). In the near future, AI will be able to stratify anxiety risk from clinical and scheduling data to suggest the most cost-effective intervention package per patient^(^11^,^37^)^. This personalization is plausible and aligns with safety and quality trends in radiology^(^4^,^7^)^.

In summary, mock scanner training, CBT, VR exposure, environmental modulation (music/aromatherapy), therapeutic communication, parental presence, and abbreviated protocols form a complementary portfolio that, when organized into scalable algorithms, reduces the need for sedation and improves clinical and operational outcomes^(^12^,^29^,^33^,^40^,^44^,^45^)^. Facilities wishing to migrate from the “sedation as the standard” model to a “sedation as the exception” model should start by conducting pilot programs with clear key performance indicators, training teams, and institutionalizing high-value bundles while constantly reassessing results and costs^(^4^,^7^,^13^)^.

## CONCLUSION

Fear of radiological examinations, especially MRI, is an underdiagnosed but highly prevalent condition with a direct impact on diagnostic quality, patient safety, and the operational efficiency of imaging clinics. This review demonstrated that there is increasing evidence that nonpharmacological strategies—such as mock scanner training, CBT, VR exposure, therapeutic communication, environmental control, and relaxation techniques—are effective in reducing anxiety, improving examination tolerance, and decreasing the need for sedation.

Despite the progress that has been made, there continue to be institutional, cultural, and financial challenges hindering the widespread adoption of these practices. Consolidating these nonpharmacological strategies as a standard of care requires institutional change and a paradigm shift, replacing the procedure-centered model with one that is patient-centered. This transformation involves revising care protocols and training multidisciplinary teams, as well as promoting an organizational culture focused on active listening, empathy, and individualized care.

We propose that healthcare institutions develop individualized management algorithms for patients with phobia related to radiological examinations, integrating prior screening, nonpharmacological techniques, and, when necessary, the judicious, safe use of sedation. Only with an integrative, evidence-based approach oriented toward the patient experience will it be possible to guarantee a technically appropriate, ethically responsible diagnostic examination that protects human dignity.

## Data Availability

The research data are available within the document.
